# Pancreaticopleural fistula and mediastinal pseudocyst: An unusual presentation of acute pancreatitis

**DOI:** 10.4103/1817-1737.33701

**Published:** 2007

**Authors:** Nagaraja Moorthy, A. Raveesha, K. Prabhakar

**Affiliations:** *Department of General Medicine, Sri Devaraj Urs Medical College, Tamaka, Kolar, India*

**Keywords:** Acute pancreatitis, mediastinal pseudocyst, pancreaticopleural fistula

## Abstract

We report a case of an adult male who presented with recurrent massive hemorrhagic pleural effusion with a mediastinal cyst due to an unusual complication of pancreatic pseudocyst-like pancreaticopleural fistula and a mediastinal pseudocyst. The clinical presentation was misleading since the patient presented with predominantly respiratory complaints. High index of suspicion of pancreatic etiology in recurrent massive hemorrhagic pleural effusion may lead to the diagnosis.

Pancreaticopleural fistula and mediastinal pseudocyst are an uncommon but significant complications of pancreatitis. Accurate and early diagnosis is dependent on recognition of characteristic clinical and imaging findings.

## Case Report

A 30-year-old male, a chronic alcoholic and smoker, was admitted to the hospital with history of fever, dry cough and pleuritic type of chest pain, progressive shortness of breath, weight loss and loss of appetite of 15 days' duration. On examination, the patient was febrile and dyspneic. Respiratory system examination was suggestive of left-sided pleural effusion. Examination of abdomen, cardiovascular system and central nervous system was normal.

Routine investigation reports were as follows: Hb - 10 g/dl, TC - 14,230 cells/cu. mm with lymphocytosis and ESR - 76 mm at 1 h. Chest X ray PA view showed left-sided massive pleural effusion. Pleural fluid was hemorrhagic with protein - 3.5 g/dl and sugar - 74 mg/dl; cell count was 720 cells/cu. mm, 60% lymphocytes, no malignant cells. Culture was sterile.

Considering the fact that tuberculosis is the most common cause of exudative pleural effusion in India; and also based on history, clinical examination and investigation reports, a diagnosis of tubercular pleural effusion was made and the patient was started on antitubercular drugs. Few days later, the patient developed marked dyspnea and a soft cystic swelling on the right side of the neck.

**Figure 1:**
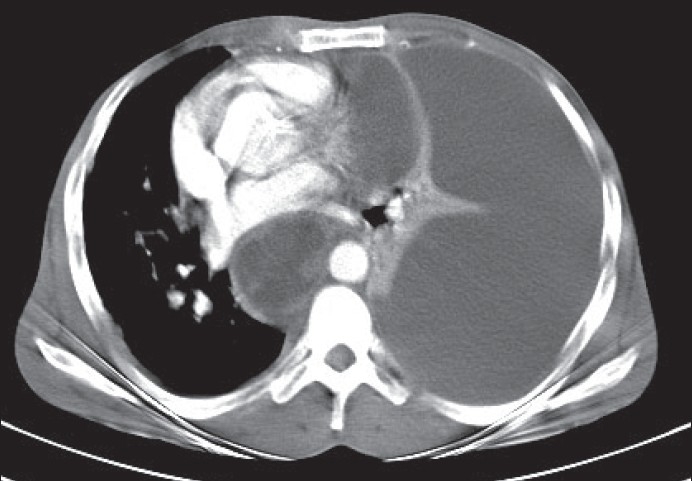
CT scan thorax showing massive left-sided pleural effusion with compressive collapse of left lung and a posterior mediastinal cyst

**Figure 2:**
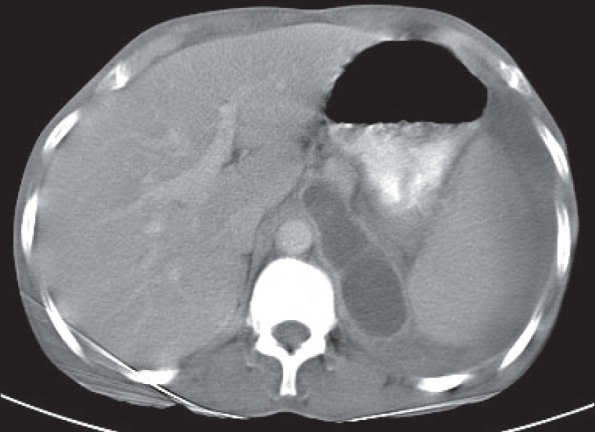
CT scan abdomen showing pancreatic pseudocyst

Computerized tomography (CT) scan thorax-abdomen showed massive left-sided pleural effusion with compressive collapse of left lung and a posterior mediastinal cyst measuring 5 × 5.5 cm just adjacent to the esophagus, extending to the neck [Figure 1]. It also showed pancreatic pseudocyst measuring 9 × 3.5 cm abutting the left crus of the diaphragm near esophageal hiatus communicating with the mediastinal cyst [Figure 2]. Pleural fluid amylase and the corresponding serum amylase were 44,671 *IU*/L and 430 *IU*/L respectively. So the diagnosis of pancreatic pseudocyst with pancreaticopleural fistula with a huge mediastinal pseudocyst was made.

The patient had recurrent episodes of massive left-sided hemorrhagic pleural effusion. He was treated with intercostal drainage for 3 weeks and intravenous octreotide. Repeat CT scan done after 3 weeks showed complete resolution of the effusion, mediastinal cyst and pseudocyst of pancreas. With the instruction to stop alcohol consumption, the patient was discharged on day 30.

Thus we have reported a case of pancreaticopleural fistula and a mediastinal pseudocyst as an unusual presentation of acute pancreatitis causing recurrent massive hemorrhagic pleural effusion, which was managed conservatively with success.

## Discussion

The more commonly recognized complications of pancreatitis are those that are localized to the abdomen, such as pancreatic and peripancreatic pseudocysts, abscess and pancreatic necrosis and pseudoaneurysm formation. However, extra-abdominal complications of pancreatitis such as thoracopancreatic fistula do occur. Thoracopancreatic fistula is a rare complication of pancreatitis that manifests as a fistulous communication between the pancreas and chest. Thoracopancreatic fistulas are divided into four types based on the termination site of the fistula - pancreaticopleural, mediastinal pseudocyst, pancreaticobronchial and pancreaticopericardial.[1]

Although the exact incidence of thoracopancreatic fistula is difficult to determine, a review of the English medical literature extending from 1965 to 1990 yielded only 89 cases of thoracopancreatic fistulas. Rockey and Cello reported the occurrence of pancreaticopleural fistulas in ∼0.4% of patients with pancreatitis. Pancreaticopleural fistulas have been noted in 2.3-4.5% of patients presenting with pancreatic pseudocyst.[2] The presentation is often confusing because of the paucity of clues suggestive of pancreatic disease and the preponderance of pulmonary signs and symptoms. Most patents are alcoholics but only one-half will have a clinical history of previous pancreatitis. Pleural effusions are large, recurrent and highly exudative in nature. Many patients go through extensive pulmonary evaluation before the pancreas is identified as the site of primary pathology.[2] The mediastinum is a relatively uncommon site for pancreatic pseudocyst. In most cases, the pseudocyst is located in the posterior mediastinum, with entry to the mediastinum via the aortic or esophageal hiatus. The most common presenting symptoms are chest or abdominal pain and dyspnea. Displacement of the esophagus and stomach anteriorly and to the left on the upper gastrointestinal series and an associated mediastinal mass are the most helpful radiographic manifestations.[3]

The diagnosis of pancreaticopleural fistula is based on the triad of massive pleural effusions, elevated pleural fluid amylase and protein levels. It may develop as a consequence of disruption of a dilated pancreatic duct. Pseudocysts are involved in the process in at least half of the reported cases.[4] Computed tomography is excellent in defining pancreatic abnormalities and should be the first abdominal imaging study in suspected cases. Endoscopic retrograde cholangiopancreatography (ERCP) is used as a diagnostic tool only in confusing cases.[2]

A substantial number of pancreaticopleural fistulas will close spontaneously utilizing conservative measures, including pancreatic rest, total parenteral nutrition and repeated thoracocentesis. Surgical correction of the underlying pancreatic disease, including ductal decompression and drainage or resection of associated pseudocysts, is indicated to prevent recurrence of the fistula and to avoid other complications of advanced chronic pancreatitis. Associated terminal biliary obstruction should be identified and managed with biliary-enteric bypass. The long-term outcome is good in 80-95% of cases.[4,5]
